# Massive Spontaneous Hemothorax as a Complication of Apixaban Treatment

**DOI:** 10.1155/2018/8735036

**Published:** 2018-10-16

**Authors:** M. Abu Hishmeh, P. Srivastava, Q. Lougheide, M. Srinivasan, S. Murthy

**Affiliations:** ^1^Lincoln Medical and Mental Health Center, Westchester Medical Center, New York Medical College, USA; ^2^Lincoln Medical and Mental Health Center, USA

## Abstract

**Introduction:**

Hemothorax is usually related to chest or iatrogenic trauma from procedures such as central lines and thoracentesis. Spontaneous hemothorax is defined as pleural fluid hematocrit greater than 50% of serum hematocrit in absence of natural or iatrogenic trauma affecting the lung or pleural space. Coagulopathy secondary to anticoagulant use has been associated with spontaneous hemothorax. We present a case of spontaneous hemothorax in a patient taking apixaban for venous thromboembolism disease. To our knowledge, this is the first case report of apixaban as a cause of spontaneous hemothorax.

**Case Presentation:**

A 56-year-old woman with end-stage renal disease (ESRD) was diagnosed with upper extremity deep vein thrombosis (DVT) one month prior to presentation and was started on apixaban presented with dyspnea and left-sided pleuritic chest pain for two weeks. She was found to have left-sided large pleural effusion which was diagnosed as hemothorax. Other etiologies for spontaneous hemothorax were excluded and drainage by 12-French pigtail catheter achieved total resolution of hemothorax in three days.

**Discussion:**

Apixaban is a DOAC used to prevent stroke or thromboembolic events in patients with nonvalvular atrial fibrillation and to prevent recurrent venous thromboembolic disease. Events such as gastrointestinal, intracranial, and soft tissue bleeding have been well-documented. However, bleeding manifestation as hemothorax is seldom reported. Our patient presented with isolated left-sided large pleural effusion which was diagnosed as spontaneous hemothorax. 12-Fr pigtail catheter drainage was effective in the management of our patient and provided total resolution in three days.

**Conclusion:**

Spontaneous hemothorax is a rare complication of anticoagulant therapy and might not exhibit the usual radiological signs of traumatic hemothorax. Health care providers should have high index of suspicion for spontaneous hemothorax when evaluating new pleural effusion in patients receiving DOACs therapy. Drainage by small bore pigtail catheter might be as effective as larger chest tubes.

## 1. Introduction

Hemothorax is defined as pleural fluid with a hematocrit > 50% of the blood hematocrit. Most cases of hemothorax are related to chest trauma or iatrogenic trauma from procedures such as central lines, thoracentesis, pleural biopsy, or catheterization [1]. Spontaneous hemothorax is less common, and the causes include malignancies, anticoagulant medications, aortic dissection, arteriovenous malformations [AVMs], endometriosis, pulmonary infarctions, and spontaneous pneumothorax [1].

Coagulopathy secondary to anticoagulant use has been associated with spontaneous hemothorax when administered in the setting of thromboembolic disease [2, 3]. Anticoagulants such as warfarin, heparin, and enoxaparin have been reported to be responsible for spontaneous hemothorax [1]. However, there are only few case reports of direct oral anticoagulants (DOACs) causing spontaneous hemothorax [4–6]. We present a case of spontaneous massive hemothorax in patient taking apixaban for venous thromboembolism disease. To our knowledge, this is the first case report of apixaban as a cause of spontaneous hemothorax.

## 2. Case Presentation

**Figure 1:**
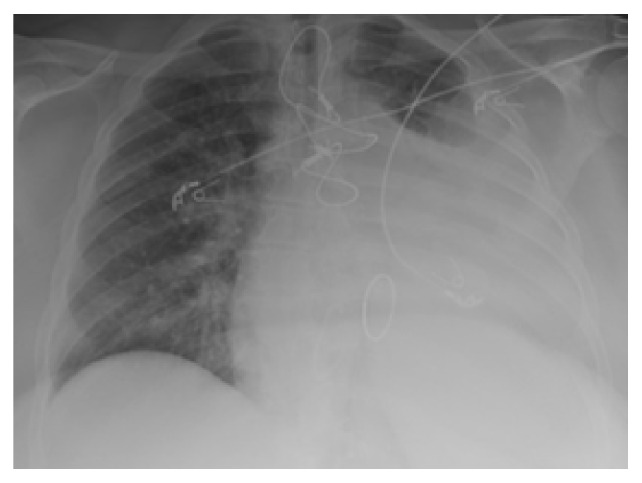
Large Left-Sided pleural effusion with tracheal deviation to the contralateral side. Also sternal wires from previous open-heart surgery are noted.

A 56-year-old woman with end-stage renal disease (ESRD) on hemodialysis (HD) was diagnosed with upper extremity deep vein thrombosis (DVT) one month prior to presentation and started on apixaban presented with dyspnea and left-sided pleuritic chest pain for two weeks that have been progressing slowly. She denied any other respiratory symptoms such as hemoptysis and cough and had no history of falls nor trauma. There was no weight loss, fever, night sweats, or recent travels. She has history of hypertension, diabetes mellitus, and prosthetic mitral valve replacement and she takes lisinopril, carvedilol simvastatin, and aspirin. Physical examination revealed tachypnea and dullness to percussion of the posterior left hemithorax along with decrease in tactile fremitus; she remained hemodynamically stable throughout her hospital stay. Laboratory tests on admission revealed the following: prothrombin time (PT) of 11.5 seconds, international normalized ratio (INR) of 1.03, activated partial thromboplastin time (aPTT) of 30.4 seconds, and hemoglobin of 7.0 g/dL. Chest radiograph (CXR) showed opacification of two-thirds of the left hemithorax with tracheal deviation to the contralateral side consistent with pleural effusion (Figure 1). Bedside ultrasound of left hemithorax revealed hypoechoic fluid without loculations. Even though she was transfused one unit of packed red blood cells (PRBC), her Hb dropped to 6.7 g/dL on the second day of admission.

**Figure 2:**
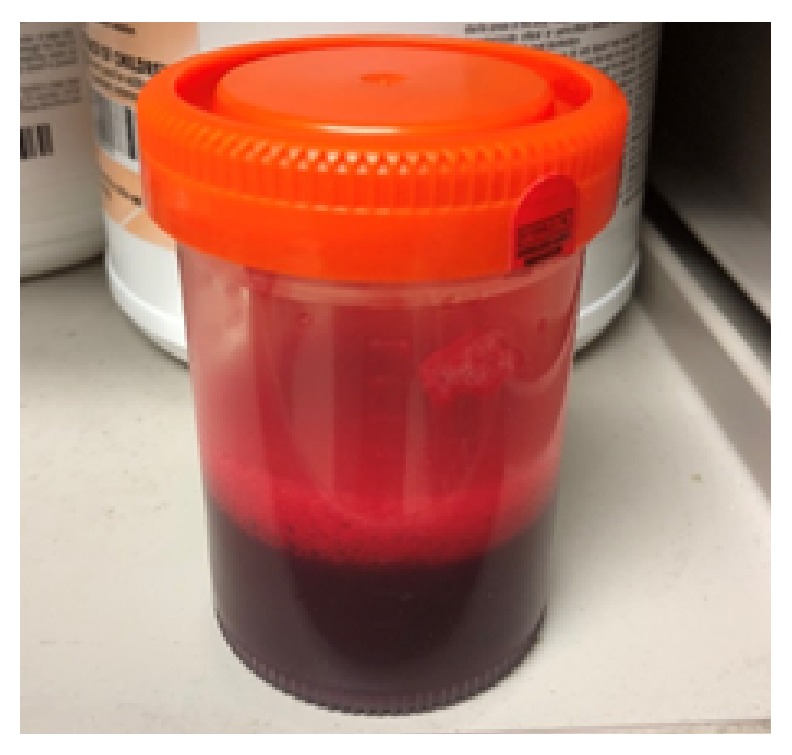
Pleural fluid sample. Of note, the whole amount of the drained fluid (1 liter) had the same color and consistency and did not clear by the end of drainage.

**Figure 3:**
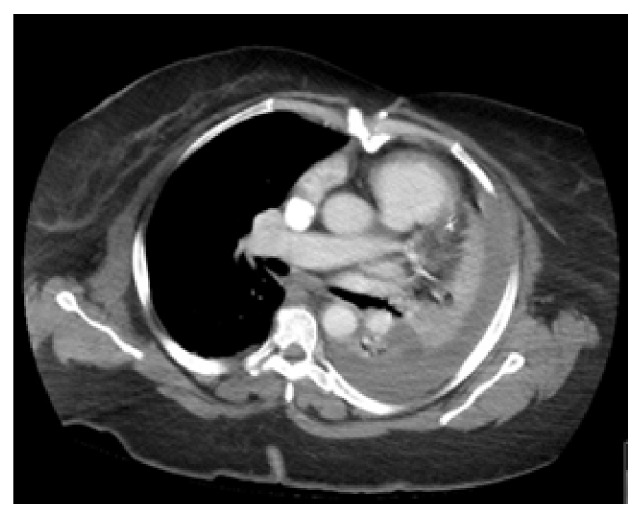
Chest CT with IV contrast after drainage of one liter. No vascular malformations, active bleeding, or other sources of hemothorax were identified. Left-sided pleural effusion was noted to have different Hounsfield unit when compared to the aorta and pulmonary artery.

Apixaban was held for 48 hours and bedside ultrasound-guided thoracentesis was performed on the third day of admission. One liter of bloody fluid was drained and sent for testing (Figure 2). Pleural fluid analysis was as follows: hematocrit (Hct) 22%, red blood cells (RBC) 126,000/mm3, white blood cells (WBC) 1,400/mm3; bacterial gram-stain, culture, and acid-fast bacilli smear were negative for bacteria and tuberculosis, respectively, and cytology negative for malignant cells. Serum Hct was 25% at the time of the thoracentesis. The finding of pleural fluid analysis was consistent with hemothorax (pleural Hct to serum Hct ratio 88%). A chest computed tomography (CT) with intravenous contrast was done to rule out active bleeding, vascular malformations, and other causes of spontaneous hemothorax (Figure 3).

**Figure 4:**
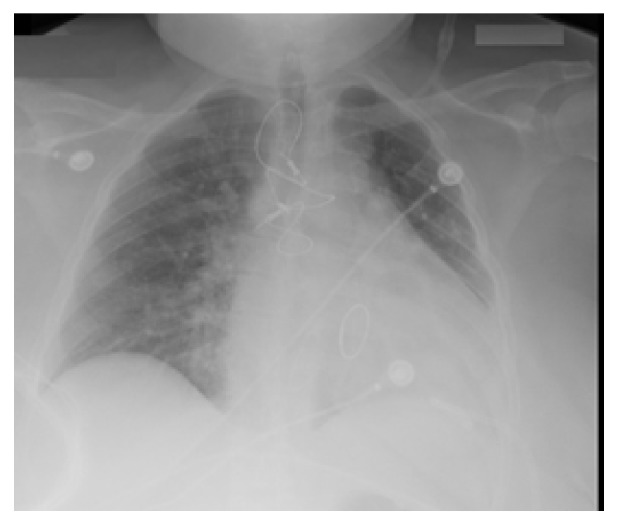
Near-total resolution of left-sided pleural effusion. Left-sided Pigtail (12-Fr) catheter and sternal wires are noted as well.

A 12-Fr pigtail catheter was inserted and another one liter was drained in the first 2 hours which then slowed down to 300ml in the next 24 hours and afterwards 20-30ml daily for 2 days. CXR after drainage showed near-total resolution of the pleural effusion (Figure 4). The catheter was then removed and patient was discharged without complications.

## 3. Discussion

Spontaneous hemothorax is defined as pleural fluid hematocrit greater than 50% of the peripheral blood hematocrit and the absence of natural or iatrogenic trauma affecting the lung or pleural space [7]. Massive hemothorax refers to a blood loss of 1500 mL [8]. Spontaneous hemothorax secondary to anticoagulant therapy usually presents in setting of pulmonary embolism early after starting the therapy and patients with pulmonary infarction are at higher risk of this complication [1–3, 9–12]. DOACs such as Rivaroxaban [4] and Dabigatran [5, 6] have been reported to cause spontaneous hemothorax in setting on pulmonary embolism and atrial fibrillation, respectively.

Apixaban is a DOAC used to prevent stroke or thromboembolic events in patients with nonvalvular atrial fibrillation and to prevent recurrent venous thromboembolic disease [13, 14]. Apixaban is the only direct oral anticoagulant (DOAC) with an FDA-approved indication for use in hemodialysis (HD) patients [15]. Events such as gastrointestinal, intracranial, and soft tissue bleeding have been well-documented. However, bleeding manifestation as hemothorax is seldom reported [13].

Our patient presented with isolated left-sided large pleural effusion raising the possibility for malignancy and Microbacterium tuberculosis infection; spontaneous hemothorax was lower in our differential diagnosis given the rarity of this condition even in coagulopathic patients without history of trauma or recent procedure. The hypoechoicity of the pleural fluid and the absence of hematocrit sign on ultrasound images were not consistent with hemothorax neither. Diagnostic thoracentesis was done after 48 hours of holding apixaban to avoid bleeding complications.

Thoracentesis was performed under ultrasound guidance and revealed bloody fluid which fulfilled diagnostic criteria of hemothorax (pleural fluid with a hematocrit > 50% of the blood hematocrit) [1]. We do not believe that the hemothorax was iatrogenic for a few reasons; firstly, fluid color and consistency remained constant during the thoracentesis and pigtail drainage and did not clear after removal of significant amount. Secondly, the patient hemoglobin remained stable after the thoracentesis and did not drop nor did the patient become hemodynamically unstable. Thirdly, the CXR showed improvement in the effusion rather than worsening and CT chest with intravenous contrast did not show signs of active bleeding.

We hypothesized that the patient has been slowly bleeding and she did not develop loculations, hematomas, or echogenicity on bedside ultrasound and CT chest because of being anticoagulated with apixaban and, in contrast to traumatic hemothorax, in patients with slower blood loss such as malignancy-associated hemothoraces, depletion of clotting factors is more pronounced; hence the usual radiological signs of hemothorax might not be present [7].

a 12-Fr pigtail catheter drainage was chosen rather than larger bore chest tube because there were no signs of loculations or hematoma on chest CT and ultrasound and for patient comfort. Bauman et al. [16] compared 14-Fr pigtail catheter with chest tube in management of traumatic hemothorax/hemopneumothorax and discovered similar outcomes in terms of complications and failure rate and the initial drainage output from pigtail catheters was not inferior to that of chest tubes. However, the usage of pigtail catheter was selective; hence they recommended further multicenter study to provide support for its usage in traumatic hemothorax and hemopneumothorax.

## 4. Conclusions

Spontaneous hemothorax is a rare complication of anticoagulant therapy and might not exhibit the usual radiological signs of traumatic hemothorax. Health care providers should have high index of suspicion for spontaneous hemothorax when evaluating new pleural effusion in patients receiving DOACs therapy. Drainage by small bore pigtail catheter might be as effective as larger chest tubes with the added benefit of patient comfort.
